# In Vitro Screening of Bacterial Isolates From Dairy Products for Probiotic Properties and Other Health‐Promoting Attributes

**DOI:** 10.1002/fsn3.4537

**Published:** 2024-11-25

**Authors:** Ishita Modasiya, Priya Mori, Hina Maniya, Mehul Chauhan, Chand Ram Grover, Vijay Kumar, Apurba Kumar Sarkar

**Affiliations:** ^1^ Postbiotics and Foodomics Lab, Department of Microbiology, School of Science RK University Rajkot Gujarat India; ^2^ Symbiotics, Functional Food and Bioremediation Lab, Dairy Microbiology Division ICAR‐N.D.R.I Karnal Haryana India; ^3^ Paradise Scientific Company Ltd Dhaka Bangladesh

**Keywords:** acid tolerance, antioxidant activity, bile tolerance, cell autoaggregation, cell surface hydrophobicity, probiotics

## Abstract

The present research was aimed to isolate potential probiotic organisms from dairy products locally made in and around the Saurashtra region of Gujarat. A total of 224 colonies were screened for primary attributes. Based on the results, 70 isolates were carried further for secondary screening. Out of these, only 23 isolates were further tested for antioxidant activities. Only 6 potential probiotic strains were found to have all the probiotic attributes. These isolates demonstrated survivability up to 4 h at pH ≤ 3, bile concentration ≥ 1.5%, autoaggregation ability ≥ 81.08%, and cell surface hydrophobicity more than 70% while using toluene as the test hydrocarbon. The promising six isolates were subjected to 16S rRNA sequencing for species‐level identification and found to be belonging to the genus Bacillus, Enterococcus, and Lactobacillus. The isolates demonstrated higher antioxidant potential as determined by ABTS, DPPH, and FRAP methods. For all three methods, *L. rhamnosus* was taken as a positive control that showed 85.61%, 39.56%, and 78.18% reduction of free radicals as determined by the ABTS, DPPH, and FRAP methods, respectively. Compared to this, *Limosilactobacillus fermentum* BAB 7912 demonstrated the highest reduction of ABTS radicals (83.45%), while Bacillus subtilis BAB 7918 reduced 29.95% DPPH free radicals and *Bacillus spizizenii* BAB 7915 reduced 80.93% ferric ions as determined by the FRAP method. Isolates were subjected to 16S rRNA sequencing for species‐level identification and found to be belonging to genus Bacillus, Enterococcus, and Lactobacillus.

## Graphical abstract

This article emphasize the isolation, characterization, and identification of indigenous probiotic isolates from the Saurashtra region of Gujarat, India, as this region has a rich culture of using fermented food in their regular diet. In this article, authors have also evaluated and screened 270 isolates for potential probiotic attributes and finally reported 6 probiotic organisms along with their 16S RNA sequences that have been submitted to the NCBI database.

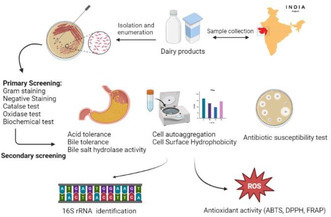

## 1 Introduction

The concept of functional food or FOSHU originated in Japan as the food for supplemental health use based on the proof of its beneficial affirmations (Sanders 1998; Lin 2003). Although functional foods' implications are well known, the COVID pandemic brought them to the public's notice because of their positive health effects. Despite the lack of a clear definition for this category of foods, functional foods may be characterized as those that include bioactive substances that provide extra health benefits beyond their nutritional value. It contributes a lot to boost immunity, and consequently the health condition, quality of life, and physical well‐being (Bhat and Bhat 2011). However, new terms like “paraprobiotics” (dead/inactivated cells of probiotics) and “postbiotics” (helpful metabolites of probiotics) have been added to the probiotic terminology in recent years (Zendeboodi et al. 2020). Probiotic microorganisms isolated from milk or its product also qualify for the FOSHU because they furnish health benefits (Abdelazez et al. 2018). It can paradoxically increase the adhesion and invasion capability of microbial pathogens in the intestinal epithelium and thus improve the mucosal barrier and immunity (Stavropoulou and Bezirtzoglou 2020). Most probiotics are lactic acid bacteria (LAB) from the *Lactobacillus* and *Bifidobacterium* genera. *Bacillus, Lactococcus, Leuconostoc, Pediococcus, Enterococcus,* and *Streptococcus* strains are among the others. They are predominantly found in the human gut microbiota, feces, breast milk, fermented foods, and dairy products (James and Wang 2019). *Lactobacillus* strains isolated from dairy products gain interest due to their huge potential for commercial product formulation along with health‐promoting properties (Amraii et al. 2014). Numerous studies have reported health benefits by consumption of probiotics at concentrations ranging between 10^8^ and 10^11^ colony‐forming units (cfu) per day (FAO/WHO Working Group 2002; Nath et al. 2020). They do so by releasing certain metabolites like bioactive peptides, short‐chain fatty acids, vitamins, and amino acids that can be beneficial for gut health by altering the inhabitants of various microbiota of the intestine (Yue et al. 2020). The therapeutic value of probiotics in ameliorating obesity, insulin resistance, hepatic steatosis, and other lifestyle diseases has also been established by several in vivo and clinical research, although it is confirmed that strains and species of probiotics have a significant impact on these beneficial effects on health (Ji et al. 2019; Kim et al. 2018; Mallappa et al. 2012). It is anticipated that probiotics extracted from regional sources could be more safe and reliable for local users since human gut flora is location‐dependent (Nagpal et al. 2018). This also establishes ground for personalized medication and therapeutic approaches.

Based on the significance of probiotics in health improvement as well as considering their potential importance in the development of food products, our study aimed to obtain and explore the bacterial isolates from the dairy products for their probiotic properties and other health‐promoting attributes.

## 2 Materials and Methods

### 2.1 Isolation and Characterization of Isolates

Isolation of potential probiotic culture was carried out by screening of traditional dairy products, including curd, buttermilk, and cow milk. Samples from different dairy stores in the local region of Gujarat were collected in sterile falcon tubes. Afterward, the collected samples were transferred and stored at the laboratory of the Department of Microbiology, RK University, Rajkot, till further analysis. One gram of each sample was dissolved in 9 mL of 0.85% NaCl (w/v), further serially diluted (10^−1^–10^−10^) and selected dilutions (10^−4^, 10^−5^, 10^−6^) were poured in presterile deMan Rogosa Sharpe (MRS) agar plates, followed by incubation for 24 h at 37°C under CO_2_‐enriched environment in a candle jar (AlKalbani, Turner, and Ayyash 2019). Isolated colonies were selected and sub‐cultured on freshly prepared and sterile MRS broth to get the pure cultures. Selected colonies were subjected to a primary screening that involved morphology of the colony, Gram staining, negative staining, oxidase, catalase, and other biochemical tests. The isolates were also tested for safety aspects via experiments like hemolytic activity (Yasmin et al. 2020).

### 2.2 Screening of Probiotic Attributes of Isolates

#### 2.2.1 Test for Acid Tolerance

An in vitro evaluation of isolates' ability to survive in the acidic environment of the colon is an essential component of probiotic culture identification. Given that food stays in the stomach for at least three hours after eating, our study's pH tolerance test was conducted throughout this time period. Instead of taking the OD, which varies due to both live and dead cells present in the media, the more reliable method for determining the viability, that is, indigenously developed spot test method, was applied to determine their survivability/tolerance for pH 1.5, 2, 3, 4, and 6.8. Briefly, 1% v/v overnight grown culture (turbidity equivalent to a 0.5 McFarland standard, which equates to approx. 1 to 2 × 10^8^ cfu/mL) was inoculated into sterile MRS broth pre‐adjusted to different pHs (1.5, 2, 3, 4, and 6.8) using 1 N hydrochloric acid (HCl) and incubated for 24 h at 37°C under microaerophilic conditions. From this broth, 1% v/v inoculum was transferred into another set of sterile MRS broth preadjusted to different pHs (1.5, 2, 3, 4, and 6.8) and incubated at 37°C for 4 h. Intermittently, 5 μL bacterial culture was spotted on the MRS agar plates from 0th h till 4th h at a regular interval of 1 h in order to determine the viability of bacteria under given pH conditions. The growth patterns of our isolates were compared with those of *Lb. plantarum* NCDC 347 and *Lb. rhamnosus* NDRI 184, while uninoculated MRS broth was taken as a negative control. The growth was measured by observing visible colonies.

#### 2.2.2 Test for Bile Tolerance

Bile tolerance of isolates obtained during the present study was evaluated by inoculating 1% v/v active culture (turbidity equivalent to a 0.5 McFarland turbidity standard, culture containing approx. 1 to 2 × 10^8^ cfu/mL) in a new MRS broth containing 0.5%, 1%, 1.5%, and 2% bile salts for enrichment. Isolates were also inoculated in MRS broth without bile salts, which work as a control. All the inoculated tubes were kept in incubation for 24 h at 37°C under microaerophilic conditions. The enriched cultures were reinoculated at 1% v/v concentration to the new MRS broth containing 0.5%, 1%, 1.5%, and 2% bile salts and MRS broth without bile salts. We followed the spot plate method, as mentioned earlier, to determine the viability of cultures.

### 2.3 Determination of Autoaggregation Activity

The potential of the LAB isolates for autoaggregation was examined following the protocol of Li et al. (2020) and Somashekaraiah et al. (2019) with minor alteration. Overnight grown cell culture (turbidity equivalent to a 0.5 McFarland turbidity standard, culture containing approx. 1 to 2 × 10^8^ cfu/mL) was centrifuged at 6000 for 10 min at room temperature for separating the pellet and supernatant. After separation, the supernatant was removed and the pellet was resuspended in phosphate buffer (PBS‐pH 6.6) and washed twice. The cell suspension was diluted using PBS to achieve an OD between 0.5–0.6 at 600 nm. The suspension so obtained was checked for change in OD at 600 nm with hourly interval till 5 h by mixing 0.1 mL of cell suspension in 3.9 mL PBS for obtaining the autoaggregation results and detecting the absorbance. The percent autoaggregation was calculated using the below‐mentioned equation. *A* = (1—OD_final_/OD_initial_) × 100%, where *A* = autoaggregation, OD_initial_ = cell suspension OD before incubation, and OD_final_ = cell suspension OD at 5th h.

### 2.4 Determination of Cell Surface Hydrophobicity

To estimate the hydrophobicity of isolated lactic cultures, the microbial adhesion to hydrocarbons (MATH) method (dos Santos et al. 2021) was followed with slight modification. The cell pellet was obtained as mentioned previously. After the pellet was washed two times with phosphate urea magnesium (PUM) buffer of pH 6.6, the cells were resuspended in PUM buffer and set to an optical density of 0.7–0.9 at 610 nm. We used two hydrocarbons (xylene and toluene) for the determination of hydrophobicity. Adjusted cell suspension was mixed with hydrocarbons in a 3:1 ratio (v/v) and vortexed for 60 s before incubating for 10 min at 37°C. For phase stabilization and separation, the mixture was vortex again and incubated at 37°C for 1 h. The optical density of the aqueous layer was observed at 610 nm. The following formula was used to compute the percentage of microbial adhesion to hydrocarbons: *H*% = (OD_initial_—OD_final_)/ OD_initial_ × 100, where *H* is hydrophobicity.

### 2.5 Determination of Bile Salt Hydrolase Activity

The qualitative direct plate assay was used for evaluating the capacity of probiotic strains for bile salt deconjugation (Kumar, Grover, and Batish 2012). Initially, 0.5% (w/v) bile slats (sodium deoxycholate and sodium taurocholate) and 0.037% calcium chloride were dissolved in water. The above mixture was added to MRS media, and plates were prepared. Activated cultures were streaked on the modified MRS agar plates and incubated at 37°C for 72 h. As a control, we used the normal MRS plates. Positive reactions were characterized by the development of an opaque halo or transparent zone surrounding the colonies due to precipitation of bile acid around the colonies.

### 2.6 Antibiotic Susceptibility Test

The disk diffusion screening technique was used to determine antibiotic susceptibility against five different antibiotics, namely ciprofloxacin, erythromycin, penicillin, tetracycline, and vancomycin (Bazireh et al. 2020). For this assay, overnight‐grown active culture having OD equivalent to 0.5 McFarland was spread on pre‐sterile MRS plates followed by the placement of antibiotic discs at equal distance. The isolates were allowed to grow in the presence of antibiotics for 24 h at 37°C in a microaerophilic condition. Bacterial susceptibility to antibiotics was evaluated by measuring the diameter (in millimeters) of the zone of inhibition surrounding the discs as per the Clinical and Laboratory Standards Institutes (CLSI) guidelines (Wayne 2017).

### 2.7 16S rRNA Identification of Selected Isolates

Following the findings of primary and secondary screening, six reference isolates were selected for 16S rRNA sequencing. For that, isolates were grown in MRS broth for 24 h and checked for purity. The selected reference strains were submitted to Gujrat Biotechnology Research Centre (GBRC), Gujrat (India) for 16S rRNA identification, and sequence data were deposited to the National Centre for Biotechnology Information (NCBI) Genbank with accession numbers OP846616, OP846617, OP846618, OP846619, OP846620, OP846621, OP846622, and OP846623, respectively. The neighbor‐joining approach was used to infer the evolutionary history (Saitou and Nei 1987). The branches are accompanied by the percentage of duplicate trees in which the related taxa grouped during the bootstrap test (1000 repetitions) (Felsenstein 1985). The evolutionary distances, which were measured in base substitutions per site, were calculated using the maximum composite likelihood approach (Tamura, Nei, and Kumar 2004). There were 17 nucleotide sequences in this analysis. For each sequence pair, all unclear places were eliminated (pairwise deletion option). There were a total of 990 positions in the final dataset. Evolutionary analyses were conducted in MEGA11 (Tamura, Stecher, and Kumar 2021).

### 2.8 Determination of in Vitro Antioxidant Activities of Isolates

#### 2.8.1 Preparation of Cell‐Free Extract

The cell‐free extract (CFE) was prepared as per the protocol of Afify et al. (2012). Briefly, sterile MRS broth was inoculated with 1% (v/v) of the overnight‐grown isolates and incubated at 37°C for 18 h. The CFE was obtained by centrifugation of the overnight‐grown isolates at 10,000 rpm for 5 min at 4°C. The CFE of isolates was subjected to different antioxidant assays, namely FRAP, ABTS, and DPPH free radical scavenging assays.

### 2.9 Ferric‐Reducing Antioxidant Potential (FRAP) Assay

The FRAP assessment was conducted with minor alteration to the method reported by Muniandy, Shori, and Baba (2016) and Ramalho et al. (2019). In a ratio of 10:1:1, 300 mM acetate buffer, 8 mM 2,4,6‐tri(2‐pyridyl)‐s‐triazine (TPTZ) (dissolved in 40 mM of HCl) reagent, and 20 mM FeCl_3_ solutions were supplemented to afresh prepare a functional FRAP reagent. Each CFE of individual isolates was examined for FRAP assay. 50 μL of CFE (sample) was added into the test tube containing 1.5 mL of freshly prepared FRAP reagent, followed by the addition of ultra‐pure water to make up the volume up to 1.7 mL. The same ultrapure water was used as a blank. Before measuring the absorbance at 593 nm in the UV spectrophotometer (Shimadzu‐UV1900), the solutions were placed in a dark condition for incubation at 37°C in the water bath for 10 min. Known concentrations of ferrous sulfate (25 μM, 50 μM, 75 μM, 100 μM, 200 μM, and 400 μM) were used to prepare the standard curve. Calculation for determining the reducing power was made as per Youn et al. (2019).

### 2.10 2, 2 Azino‐Bis (3‐Ethyl‐Benzothiazoline) 6‐Sulfonic Acid (ABTS) Assay

ABTS (2, 2 azino‐bis (3‐ethyl‐benzothiazoline) 6‐sulfonic acid) activity was determined using the ABTS radical cation method (Re et al. 1999) with some modifications described by Rossini et al. (2009). A stock solution of ABTS was made using 7 mM ABTS in PBS (pH 7.4) and 2.45 mM potassium persulfate (final concentration) kept at room temperature for 14–16 h in the dark. The pre‐activated ABTS solution was diluted with PBS to obtain and optical density of the solution equivalent to 0.70 ± 0.02 for the reaction, which was further used for the assay. 990 μL of pre‐activated ABTS solution was allowed to react with 10 μL of CFE, and change in OD was measured at 734 nm at a 30‐s interval up to 6 min. The initial and finale OD was used to calculate the percentage of radical scavenging activity [*A*_734_ initial − *A*_734_ finale/*A*_734_ initial] × 100 (Re et al. 1999; Pieniz et al. 2014). Ascorbic acid was used as a standard (100–1000 μM) to plot the standard curve.

### 2.11 2, 2‐Diphenyl‐1‐Picrylhydrazyl (DPPH) Assay

The DPPH (2, 2‐diphenyl‐1‐picrylhydrazyl) radical scavenging activity was evaluated using the methods of Bondet, Brand‐Williams, and Berset (1997) and Pieniz et al. (2014). DPPH free radical solution of 5 mM strength was prepared by dissolving 9.8 mg DPPH in 5 mL 70% methanol, of which the OD at 515 nm was set to 0.70 ± 0.02. In 980 μL of the working DPPH solution, 20 μL of CFE was added, and absorbance was measured immediately for 6 min at a 30‐s interval using a UV spectrophotometer (Shimadzu‐UV1900). The percentage of radical scavenging activity was calculated according to the equation [*A*_515_ initial − *A*_515_ finale/*A*_515_ initial] × 100 (Re et al. 1999; Pieniz et al. 2014). To plot a standard curve, ascorbic acid was used as a standard ranging from 100–1000 μM.

### 2.12 Statistical Analysis

The analysis of variance (ANOVA) by Tukey test was used to evaluate the data, and statistical comparisons and analyses were carried out using one‐way and two‐way analysis of variance (ANOVA) by Tukey test with the help of GraphPad Prism version 8. The difference between the means was compared using a one‐way and two‐way ANOVA with a significant level of *p* < 0.05. All assays were performed in three independent experiments, and results are expressed as mean ± standard deviation (SD), taking n equals to three.

## 3 Results and Discussion

### 3.1 Isolation and Characterization of Lactic Acid Bacteria From Traditional Dairy Products

Various studies have established that continuous use of probiotics in everyday diets leads to health‐promoting effects. It includes improved gut microbiota and control over non‐communicable diseases like obesity, hypersensitivity, cardiovascular diseases, etc. (Yoo and Kim 2016). Dairy products are rich sources of probiotics. A variety of probiotic microbes have been isolated from milk and dairy products. Hence, our study aimed at the isolation of potential probiotic strains from the indigenous dairy product and evaluating potential probiotic isolates for secondary attributes like acid tolerance, bile tolerance, cell surface hydrophobicity, cell autoaggregation, and antibiotic susceptibility. As a positive control, *Lb. plantarum* NCDC 347 and *Lb. rhamnosus* NDRI 184 obtained from the ICAR‐National Dairy Research Institute with proven probiotic properties were used. We explored 120 dairy samples, including curd (60), milk (43), buttermilk (15), and yogurt (2), to obtain 224 isolates on the basis of their appearance, colony morphology, Gram staining, and negative staining. Out of these, 70 isolates were further selected for screening for secondary attributes. Based on the results, only six isolates were considered as potential probiotic organisms (results are presented in Tables S1 and S2) and identified by 16S rRNA sequencing. These six isolates were identified as *B. spizizenii* BAB 7915, *B. subtilis* BAB 7911, *B. subtilis* BAB 7918, *Lb. fermentum* BAB 7912, *Ent. faecalis* BAB 7913, and *Ent. faecium* BAB 7914. Primary screening data of these selected isolates along with standard probiotic cultures (*Lb. plantarum* NCDC 347 and *Lb. rhamnosus* NDRI 184) are presented in Tables S1 and S2.

### 3.2 Screening of Isolates for Probiotic Attributes

#### 3.2.1 Acid Tolerance

The Indian Council of Medical research (ICMR) has framed guidelines and a set of attributes that an isolate shall have to be called as probiotic. There are four crucial properties for the selection of strains as a probiotic. Primarily, the isolates shall thrive in varying acidic conditions and tolerate the bile salts in the human intestine (Ganguly et al. 2011; Kandylis et al. 2016; Ayyash et al. 2018). The pH of the stomach is acidic due to secretions like hydrochloric acid, sodium chloride, and potassium chloride secreted by the cell lining of the stomach. It takes approximately 4 h for food to be digested in the human stomach. Probiotics make the transient microflora of the human intestine; thus, the potential isolates must adhere, survive, and multiply on the intestinal lining in order to confer health benefits (Fujimori 2020). Hence, it becomes essential to check the survivability of isolates under low pH conditions for a longer time. We selected the pH range (1.5, 2, 3, 4, and 6.8) that mimics the gastrointestinal tract (GIT) environment. The strain's ability to survive under acidic pH environment up to 4 h at 37°C was measured, the outcomes of which are presented in Table 1. 33% of our isolates demonstrated survivability between pH 1.5 and 3 up to 4 h in the form of viable growth on modified MRS media. *Lb. fermentum* BAB 7912, *Ent. faecium* BAB 7914 showed ability to grow at pH 1.5 and 2 up to 4 h of incubation, while, at pH 2, four isolates (*Bacillus spizizenii* BAB 7915, *B. subtilis* BAB 7911, *B. subtilis* BAB 7918, and *Ent. faecalis* BAB 7913) were able to survive along with the two standards as shown in Table 1. Previous investigations have reported that probiotic bacteria that could tolerate pH 3.0 have greater survivability under gut conditions (Hosono 1999; Shokryazdan et al. 2014). The isolates were further checked for bile tolerance.

**TABLE 1:** *In vitro* acid tolerance and bile tolerance of isolates compared to standard strains *Lactiplantibacillus plantarum* NCDC 347 and *Lacticaseibacillus rhamnosus* NDRI 184.

Cultures	Time (h)	Growth at different pH					Growth at different bile concentrations (w/v %)				
		6.8	4.0	3.0	2.0	1.5	0	0.5	1.0	1.5	2.0
*Lactiplantibacillus plantarum* NCDC 347	0	+++	+++	+++	+++	+	+++	+++	+++	+++	+++
	1	+++	+++	++	++	+	+++	+++	+++	+++	+++
	2	+++	+++	++	++	+	+++	+++	+++	+++	+++
	3	+++	+++	++	++	+	+++	+++	+++	+++	+++
	4	+++	+++	++	++	+	+++	+++	+++	+++	+++
*Lacticaseibacillus rhamnosus* NDRI 184	0	+++	+++	++	++	+	+++	+++	+++	+++	+++
	1	+++	+++	++	+	+	+++	+++	+++	+++	+++
	2	+++	+++	++	+	+	+++	+++	+++	+++	+++
	3	+++	+++	++	+	+	+++	+++	+++	+++	+++
	4	+++	+++	++	+	+	+++	+++	+++	+++	+++
*Bacillus spizizenii* BAB 7915	0	+++	+++	+++	+++	+	+++	+++	+++	+++	+++
	1	+++	+++	+++	+++	−	+++	+++	+++	+++	+++
	2	+++	+++	+++	+++	−	+++	+++	+++	+++	+++
	3	+++	+++	+++	+++	−	+++	+++	+++	+++	+++
	4	+++	+++	++	++	−	+++	+++	+++	+++	+++
*B. subtilis* BAB 7911	0	+++	++	++	+	−	+++	+++	+++	+	+
	1	+++	++	++	+	−	+++	+++	+++	+	+
	2	+++	++	++	+	−	+++	+++	+++	+	+
	3	+++	+	+	+	−	+++	+++	+++	+	+
	4	++	+	+	+	−	+++	+++	+++	+	+
*B. subtilis* BAB 7918	0	+++	+++	+++	++	−	+++	+++	+++	+++	+++
	1	+++	+++	+++	+	−	+++	+++	+++	+++	+++
	2	+++	+++	+++	+	−	+++	+++	+++	+++	+++
	3	+++	+++	+++	+	−	+++	+++	+++	++	+++
	4	+++	++	−	−	−	+++	+++	+++	++	++
*Lb. fermentum* BAB 7912	0	+++	+++	+++	++	++	+++	+++	+	+	+
	1	+++	+++	+++	++	++	+++	+++	+	+	+
	2	+++	+++	+++	++	++	+++	+++	+	+	+
	3	+++	+++	+++	++	+	+++	+++	+	+	+
	4	+++	+++	+++	++	+	+++	+++	+	+	+
*Ent. faecalis* BAB 7913	0	+++	+++	+	−	−	+++	+++	+++	+++	+++
	1	+++	+++	+	−	−	+++	+++	+++	+++	+++
	2	+++	+++	+	−	−	+++	+++	+++	+++	+++
	3	+++	+++	+	−	−	+++	+++	+++	+++	+++
	4	+++	+++	+	−	−	+++	+++	+++	+++	+++
*Ent. faecium* BAB 7914	0	+++	+++	++	++	+	+++	+++	+++	+++	+++
	1	+++	++	++	+	+	+++	+++	+++	+++	+++
	2	+++	++	++	+	+	+++	+++	+++	+++	+++
	3	+++	++	++	+	+	+++	+++	+++	+++	+++
	4	+++	++	++	+	+	+++	+++	+++	+++	+++

*Note:* Data presented here are qualitative and are based on the results of experiments performed in triplicate. The number of “+” represents the noticeable growth as visible to moderately visible to transparent and “−” represents no growth of isolate on MRS agar.

#### 3.2.2 Bile Tolerance

The cell wall of microorganisms consists of lipids and fatty acids. Bile salts can destabilize them in the duodenal part of the gut. As a result, the ability of probiotics to survive at moderate to high concentrations of bile salt is a crucial trait that allows them to adequately accomplish their function in the gastrointestinal tract (Jain, Mehta, and Bharti 2017; Sivamaruthi et al. 2020). For the bile tolerance assay, isolates were grown in MRS broth containing different concentrations of bile salts like 0.5%, 1%, 1.5%, 2%, and MRS broth without the salts as a control. All 70 isolates were able to tolerate 2% bile salts (data presented in Table 1). Falah et al. (2019) and Kaushik et al. (2009) also showed similar results; their strains of *Lb. fermentum* and LP9 (*Lb. plantarum*) were able to survive at 3% and 2% bile concentrations, respectively. The results were verified by performing viability tests on MRS agar, where all isolates displayed visible colonies. The survival rate of isolates in an acidic environment and under high bile concentration varied significantly in this investigation, as observed in the growth pattern of a few cases where the isolates were tolerant to higher bile salt concentrations but were unable to survive at pH as low as 1.5 or even pH 2. This could be related to the fact that acid and bile tolerance mechanisms differ by species and strains (Nami et al. 2019).

### 3.3 Cell Autoaggregation

**FIGURE 1:**
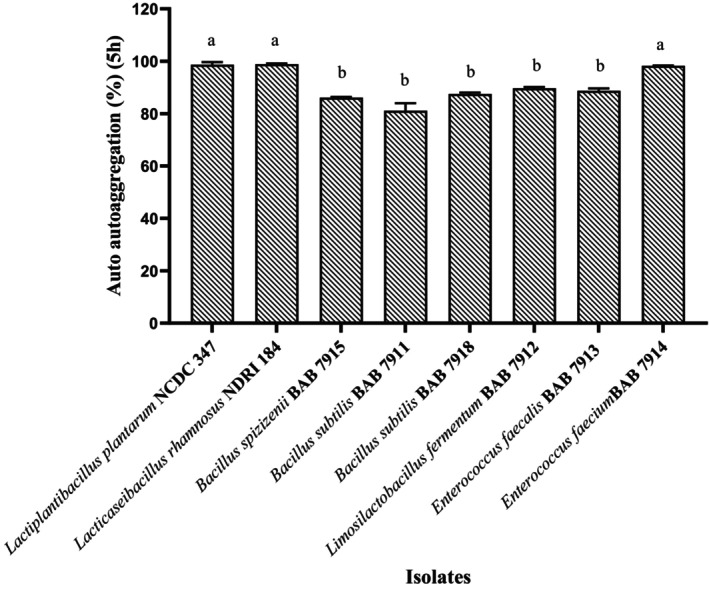
In vitro cell autoaggregation of isolates. Data are expressed as % of autoaggregation measured after 5 h of incubation. Values are presented as Mean ± SD. ^abcd^ *p* < 0.05: Significantly different. Bars with common superscripts represent non‐significance in the property.

The ability of a bacterial species to colonize the gut is one of the essential features that determined the gut microflora composition. As the probiotics constitute transient microflora, it is essential that they colonize the gut lining and persist for longer time in order to confer health promoting effects (Kaushik et al. 2009; Nikolic et al. 2012). Proteins, soluble proteins, carbohydrate, and lipoteichoic acid are examples of bacterial cellular surface components that are engaged in intracellular interaction, leading to cellular aggregation within or between species/genus (Kumara et al. 2019). The seventy isolates obtained in the present study were examined for autoaggregation activity. Results are presented in Figure 1 for six isolates that were able to survive at lower pH and higher bile salt concentrations. The results revealed that our isolates had autoaggregation ability ranging from 81.0 ± 2.8% to 98.9 ± 0.2%. The two standard cultures *Lb. plantarum NCDC 347* (98.7 ± 1.0%) and *Lb. rhamnosus* NDRI 184 (98.9 ± 0.2%) and *Ent. faecium* BAB 7914 (98.3 ± 0.0%) showed higher autoaggregation ability, while the lowest autoaggregation ability was reported for *B. subtilis* BAB 7911 (81.0 ± 2.8%). Data presented in Figure 1 (values are in Table S3) show that *Ent. faecium* BAB 7914 is significantly different (*p* < 0.0001) with all of the isolated strains and non‐significant with standard strains. Remaining isolates are not significantly different (*p* > 0.05) from each other. Byakika et al. 2020 reported that *Lb. rhamnosus* yoba 2012 and *Lb. plantarum* MNC 21 had 100% autoaggregation after 5 h incubation, and the remaining all strains had 100% autoaggregation after 24 h incubation. In our studies, the highest autoaggregation was 98% after 5 h incubation and all remaining strains had > 80% autoaggregation ability compared to the reported strains of Byakika et al. (2020). Our findings suggest the isolates of the current study are compared to previously reported findings of up to 70% autoaggregation (Espeche et al. 2012; Kos et al. 2003; Kaushik et al. 2009). Overall, the variation in ability of cell autoaggregation, which is strain‐specific and fluctuates according to factors such as the source of the bacteria, variation in the level of cell surface protein expression, and the effect of the environment on the expression of some proteins (Farid et al. 2021).

### 3.4 Cell Surface Hydrophobicity

**FIGURE 2:**
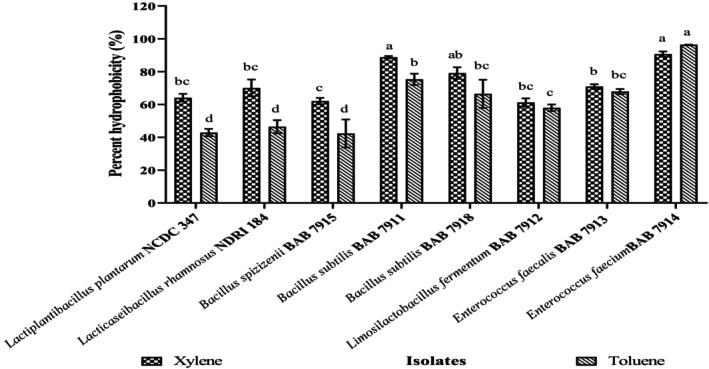
Percent cell surface hydrophobicity of isolates. Values are presented as Mean ± SD. ^abcd^ *p* < 0.05: Significantly different. Bars with common superscripts represent non‐significance in the property.

Cell surface hydrophobicity (CSH) of organisms appears to play a key role in many aspects of human health (Krausova, Hyrslova, and Hynstova 2019). For adhesiveness, a probiotic strain must possess 40% or more hydrophobicity as per Jain, Mehta, and Bharti (2017). Bacterial surfaces with higher nonpolar characteristics adhere to hydrocarbons more strongly and hence shall show higher hydrophobicity (Farid et al. 2021). It was determined against xylene and toluene by using the MATH method. These two solvents were applied to identify the best percent hydrophobicity of 70 isolates. Out of 70 isolates, the results of six isolates and two reference strains, *Lb. plantarum* NCDC 347 and *Lb. rhamnosus* NDRI 184, are presented in Figure 2 (values are in Table S3). There was a great disparity in hydrophobicity for all the test cultures, ranging from 42.3 ± 8.5% to 96.4 ± 0.1%. *Ent. faecium* BAB 7914 demonstrated strong adhesion potential of 90.7 ± 1.6% and 96.4 ± 0.1% for both the hydrocarbons xylene and toluene, respectively, while for the set of hydrocarbons, the lowest CSH was demonstrated for *Bacillus spizizenii* BAB 7915 (62.1 ± 1.9% for xylene, 42.3 ± 8.5% for toluene). We analyzed and compared the hydrophobicity attributes of isolates toward xylene and toluene separately by ANOVA. In our findings, six out of seventy isolates were showing more than 40% hydrophobicity, while four of them demonstrated more than 70%, indicating that this is a trait unique to the strain (Bhushan et al. 2021). Tyfa, Kunicka‐Styczynska, and Zabielska (2015) classified and separated bacterial strains into three categories: strongly hydrophobic (> 50%), moderately hydrophobic (20%–50%), and hydrophilic (< 20%) based on the degree of adherence to hydrocarbons. In all our findings, isolates fall into the group classified as strongly hydrophobic based on this criterion. Falah et al. (2019) showed hydrophobicity degree of *Lb. fermentum* 4–17 to be 43%, and Dhewa et al. (2010) showed hydrophobicity of *Lb. fermentum* toward xylene (62%) and toluene (56%), which is relatively near to our findings for *Lb. fermentum* BAB7912. Bacterial hydrophobicity is typically attributed to surface teichoic acids and proteins with net negative charge, although it can also be influenced by growth conditions, cell age, and environmental stress (Bhushan et al. 2021). Variations in the surface polymer composition responsible for the hydrophobic interaction, which affects the strength of adhesion, could also be the cause of fluctuations in the hydrophobicity of cell surfaces.

### 3.5 Bile Salt Hydrolase Test

One of the most common factors to evaluate probiotic strains is their ability to detoxify bile salts by activating the BSH enzyme. An enzyme called bile salt hydrolase catalyzes the deconjugation of bile salts that release free primary bile acids. Probiotics become more tolerant of bile salts due to the presence of bile salt hydrolase (BSH), which also lowers the blood cholesterol level of the host and it is varied depending on the species (Shehata et al. 2016).

The BSH activity of lactic cultures was examined by using the qualitative direct plate method against two different bile salts sodium deoxycholate (SD) and sodium taurocholate (ST), and Table 2 displays the outcomes of this test. As per Table 2, it can be observed that BSH activity was detected for two reference strains (*Lb. plantarum* NCDC 347 and *Lb. rhamnosus* NDRI 184) and only two isolated strains (*Lb. fermentum* BAB 7912 and *Ent. faecium* BAB 7914), while the remaining four strains (*B. spizizenii* BAB 7915, *B. subtilis* BAB 7911, *B. subtilis* BAB 7918, and *Ent. faecalis* BAB 7913) were able to grow on bile‐salt supplemented MRS plates, but there was no detection of zone surrounding the colonies. Previous studies have reported that *Lb. fermentum* shows higher BSH activity and absorbs cholesterol. Yerlikaya and Akbulut (2020) reported that some of the strains of *Ent. faecium* grew in the presence of taurocholic acid and glycocholic acid but did not form deconjugation, while other isolates of their study were not able to grow in the presence of sodium deoxycholate. They also reported *Enterococcus* species were more susceptible to SD in this regard. The majority of *Enterococcus* strains deconjugated sodium taurocholate only. As compared to the findings of Yerlikaya and Akbulut (2020), our isolated *Enterococcus* strains were deconjugated ST and also SD.

**TABLE 2:** Screening of lactic cultures for bile salt hydrolase activity against sodium deoxycholate (SD) and sodium taurocholate (ST).

Sr. No.	Isolates	Zone of bile slats	
		SD	ST
1.	*Lb. plantarum* NCDC 347	+	+
2.	*Lb. rhamnosus* NDRI 184	+	+
3.	*B. spizizenii* BAB 7915	−	−
4.	*B. subtilis* BAB 7911	−	−
5.	*B. subtilis* BAB 7918	−	−
6.	*Lb. fermentum* BAB 7912	+	+
7.	*Ent. faecalis* BAB 7913	−	−
8.	*Ent. faecium* BAB 7914	+	+

*Note:* Data presented here are qualitative and are based on the results of experiments performed in triplicate. “+” represents zone and “−” represents only growth without zone.

### 3.6 Antibiotic Susceptibility Test

Since antibiotic‐resistant LAB strains can contribute as possible carriers of antimicrobial resistance genes and pass them to bacterial pathogens, the determination of the antibiotic susceptibility of LAB strains is critical from a safety standpoint for their usage as prospective probiotics. According to the Scientific Committee on Animal Nutrition (SCAN) of the European Union (EU), microbes used in food and feeds should not have gained antibiotic resistance (Sharma, Sharma, and Sharma 2016). The disc diffusion method, as suggested by the Clinical and Laboratory Standards Institute, was used to investigate the pattern of resistance/susceptibility toward antibiotics of selected six isolates keeping two standard probiotics. Findings of our experiment are presented in Table 3. *Ent. faecalis* BAB 7913, *Lb. fermentum* BAB 7912, and standard probiotic strains *Lb. plantarum* NCDC 347 and *Lb. rhamnosus* NDRI 184 were resistant to vancomycin. On the other hand, *B. spizizenii* BAB 7915, *B. subtilis* BAB 7911, *B. subtilis* BAB 7918, and *Ent. faecium* BAB 7914 were sensitive/intermediate sensitive to all five antibiotics. Some LAB have an innate resistance to antibiotics. Among the processes responsible are lack of target, limited permeability, drug inactivation, and the existence of efflux systems (Byakika et al. 2020). A few antibiotic resistance LABs may serve as a gene reservoir for antibiotic resistance that might get propagated to pathogens (Mathur and Singh 2005). The risk of resistance gene transfer is, however, not only hypothetical but also practically nonexistent with inherent resistance (Broaders, Gahan, and Marchesi 2013). As an example, it is widely accepted that vancomycin resistance exists in the majority of *Lactobacilli* sp. Vancomycin targets D‐Ala‐D‐lactate in the cell wall; consequently, this resistance is specific to those cells and cannot be transferred to other cells (Byakika et al. 2020). Thus, the outcome of isolated lactobacilli strains and reference strains could be explained by the inherent vancomycin resistance, and these outcomes concur with those of Leite et al. (2015).

**TABLE 3:** Antibiotic susceptibility pattern of isolates with standard strains *Lactiplantibacillus plantarum* NCDC 347 and *Lacticaseibacillus rhamnosus* NDRI 184.

Sr. No.	Isolates	CI_5_	ER_15_	PE_2_	TE_30_	VA_30_
1.	*Lb. plantarum* NCDC 347	IS	IS	S	IS	R
2.	*Lb. rhamnosus* NDRI 184	IS	S	IS	S	R
3.	*B. spizizenii* BAB 7915	S	IS	IS	S	IS
4.	*B. subtilis* BAB 7911	S	IS	IS	IS	IS
5.	*B. subtilis* BAB 7918	S	S	S	IS	IS
6.	*Lb. fermentum* BAB 7912	IS	IS	IS	IS	R
7.	*Ent. faecalis* BAB 7913	IS	IS	IS	IS	R
8.	*Ent. faecium* BAB 7914	S	IS	IS	IS	IS

*Note:* Inhibition zones: S = 16–26 mm; IS = 3–15 mm; R = 0–2 mm.

Abbreviations: IS, Intermediate sensitive; R, Resistance; S, Sensitive; CI_5_ = Ciprofloxacin (5 μg); ER_15_ = Erythromycin (15 μg); PE_2_ = Penicillin (2 units); TE_30_ = Tetracycline (30 μg); VA_30_ = Vancomycin (30 μg). Interpretation are based on observation in triplicate.

### 3.7 16S rRNA Identification of Isolates

Based on the assessment of probiotic attributes, potential six isolates were selected for 16S rRNA identification. The results of 16S rRNA sequencing identified our isolates to be *Lactobacillus*, *Enterococcus*, and *Bacillus* species. Although *Enterococcus* sp. has not yet received the Generally Recognized as Safe (GRAS) status, in contrast to other LAB genera. Additionally, the European Food Safety Authority's Qualified Presumption of Safety (QPS) list does not recommend any *Enterococcus* sp. for human consumption. However, various researchers have reported probiotic attributes of *Enterococcus* sp. and their safety aspects, indicating the potential utility of *Enterococcus* sp. as probiotics (Nami et al. 2019; Hanchi et al. 2018; Girijakumari, Ethiraja, and Marimuthu 2018; Yerlikaya and Akbulut 2020). For any strain to be added to the functional food category, it must be evaluated for safety assessment because pathogenicity is a strain‐dependent attribute (Girijakumari, Ethiraja, and Marimuthu 2018; Hanchi et al. 2018). Hence, we conclude that our isolates that were identified as *B. spizizenii* BAB 7915, *B. subtilis* BAB 7911, *B. subtilis* BAB 7918, *Lb. fermentum* BAB 7912, *Ent. faecalis* BAB 7913, and *Ent. faecium* BAB 7914 qualify as probiotics as they are non‐pathogenic, safe for human consumption, and confer health‐promoting effects, and thus can be recommended for use as probiotics (FAO/WHO Working Group 2002)

**FIGURE 3:**
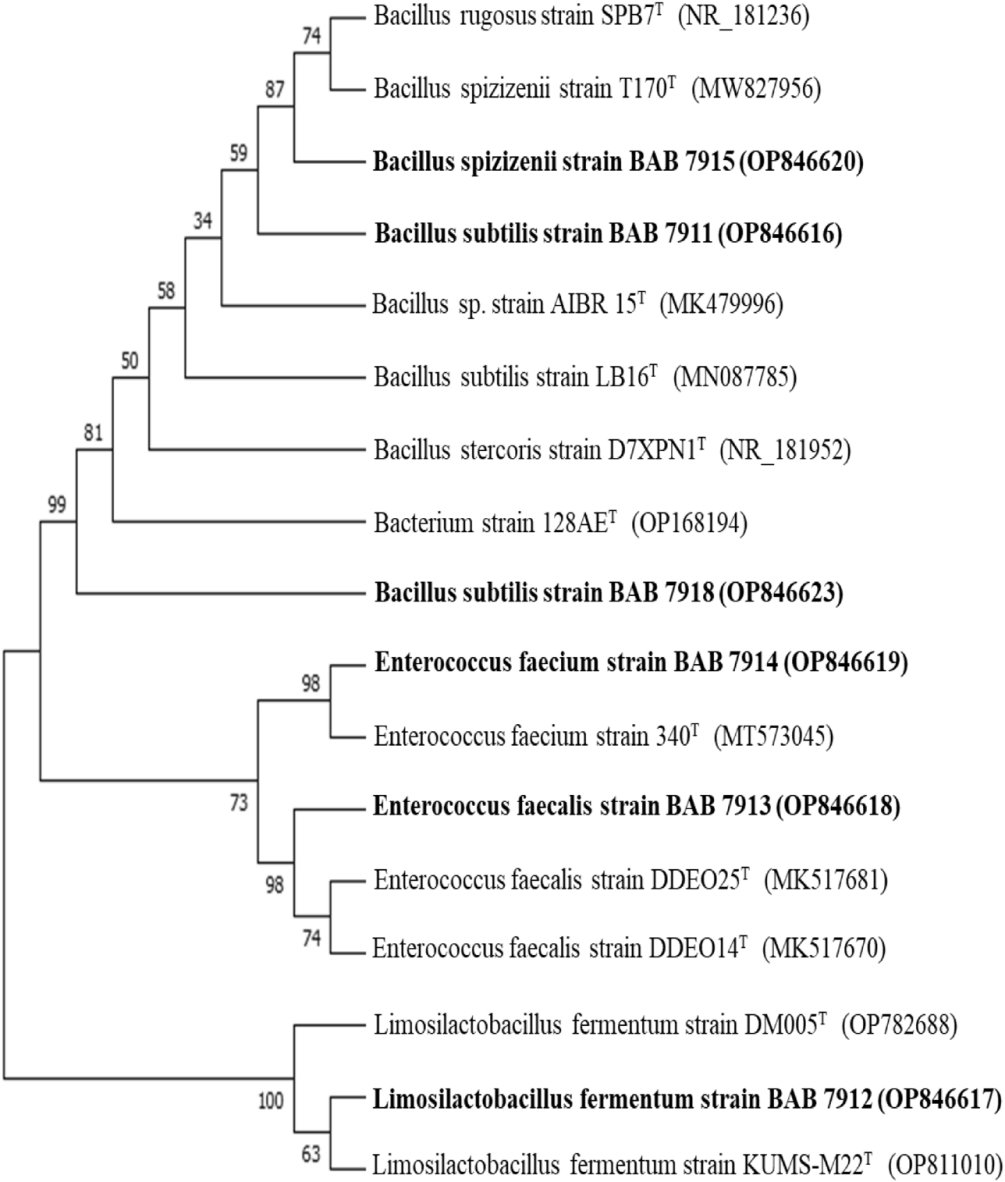
The phylogenetic tree based on 16S rRNA genes of isolates and type strains. T = Type strains.

The phylogenetic tree shown in Figure 3 indicated the relative placements of the isolates. The significant strains were classified into groups (A to C) by phylogenetic analysis. Group A, B, and C belonged to the *Bacillus* genus, *Lactobacillus* genus, and *Enterococcus* genus, respectively. A similarity search revealed that *Bacillus spizizenii* BAB 7915 and *B. subtilis* BAB 7918 were 100% similar, while *B. subtilis* BAB 7911 had 99.89% similarity with *Bacillus* genus. The fact that certain *Bacillus* sp. have been given the GRAS classification has actually received attention in them as probiotic starter cultures and food biopreservatives for the formulation of functional food with probiotic advantages like preventing and treating diarrhea, gingivitis, and *H. pylori* infection, and preserving gastrointestinal homeostasis for consumers (Dabire et al. 2022; Das et al. 2021; Elshaghabee et al. 2017). Another isolate of ours identified as *Lb. fermentum* BAB 7912 was 99.78% identical to the genus *Lactobacillus*. As probiotic products, researchers frequently utilize *Lactobacillus acidophilus*, *Lb. casei*, *Lb. paracasei*, *Lb. rhamnosus*, *Lb. delbrueckii* subsp. *bulgaricus*, *Lb. brevis*, *Lb. johnsonii*, *Lb. plantarum*, and *Lb. fermentum* (Fijan 2014; Panicker et al. 2018). *Lb. fermentum*, one of these species, is widely used in various commercialized and tribal probiotic preparations. Several processed foods utilize *Lb. fermentum* strains to improve their nutritional content, textural properties, and other attributes (Pakroo et al. 2022). Strains in group C *Ent. faecalis* BAB 7913 (99.66%) and *Ent. faecium* BAB 7914 (99.89%) were closely related to *Enterococcus* genus. The enterococcal probiotics that are now available on the market have not been linked to any illnesses. Furthermore, certain strains, such as *E. faecium* SF68 and *E. faecalis* Symbioflor, were examined for safety and have been available for over 20 years without any issues being reported. This is a reliable sign that these strains are safe (Franz et al. 2011).

### 3.8 In Vitro Antioxidant Assay (FRAP, ABTS, and DPPH)

**FIGURE 4:**
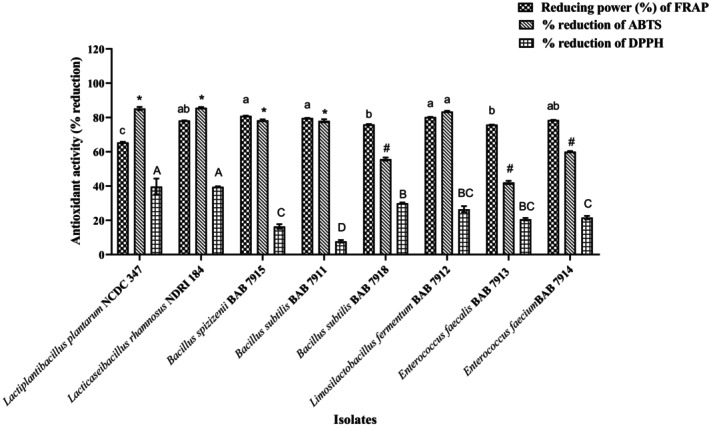
Antioxidant potential (FRAP, ABTS, DPPH) values of different isolates. Values are presented as mean ± SD. ^abcd^ *p* < 0.05: Significantly different. Bars with common superscripts represent non‐significance in the property.

In order to protect the body from free radical‐induced diseases, antioxidants play a vital role (Lee, Koo, and Min 2004). These antioxidants differ in chemical structure, mode of action, and efficiency. Hence, their activity in various dietary systems varies significantly (Moharram and Youssef 2014). Principally, there are two different mechanisms of antioxidant assay; the first method relies on hydrogen atom transfer (HAT) and the other detects electron transfer (ET). The antioxidant potential of isolates obtained in the present study was determined using three different methods: ABTS, DPPH, and FRAP. The ABTS assay is based on hydrogen atom transfer reactions, and the DPPH and FRAP assay quantifies electrons transferred during free radical scavenging reactions. The FRAP method is frequently utilized to assess the antioxidant activity of various biological samples, food materials, and plant extracts containing phenolic compounds due to its reproducibility (Dontha 2016). The potential of biological components to scavenge free radicals created is referred to as the total antioxidant capacity. All isolates demonstrated more than 75% free radical reducing power toward ferric ions (reduction of ferric ion to ferrous ion). Values are presented in Table S4. Out of these isolates, *Lb. fermentum* BAB 7912 and *B. spizizenii* BAB 7915 showed higher reducing power of 80.26% and 80.93 ± 0.12, respectively, which is more than that of standard cultures. When the ABTS method was applied to evaluate the antioxidant power, the average reducing power was found to be 28.57%, with *B. spizizenii* BAB 7915 and *B. subtilis* BAB 7911 showing below‐average ABTS reducing power (16.40% and 7.75%, respectively). The highest % reduction of ABTS was exhibited by *Lb. fermentum* BAB 7912 (83.45%). Parallelly, the DPPH (2, 2‐diphenyl‐1‐picrylhydrazyl) method was also used to access the antioxidant potential of isolates. The highest % reduction of DPPH is found in culture *B. subtilis* BAB 7918 (29.95 ± 0.40) and compared with two standard bacterial cultures, *Lb. rhamnosus* NDRI 184 (39.69%) and *Lb. plantarum* NCDC 347 (39.56%). As per the DPPH assay method, we observed a variation (*p* < 0.05) between values of the ABTS, DPPH, and FRAP assays of isolates that were examined individually. Our isolate *Lb. fermentum* BAB 7912 demonstrated the highest free radical reduction (FRAP: 80.26 ± 0.05%, ABTS: 83.45 ± 0.39%) compared to other cultures. The comparative results of our isolates for all the three methods are presented in Figure 4. The inconsistent results of three different methods may be attributed to the fact that antioxidant substances differ structurally in terms of their polarity, ionic conditions, hydrogen‐bond interaction power, solubility, and stereostructure (Ozcan et al. 2018). Similar findings for *Lb. fermentum* have been reported by Han et al. (2017). *Lb. fermentum* strains showed great efficacy, making them intriguing probiotics with possible antioxidant action for the health promotion of the host.

## 4 Conclusion

The present population of India consists of a mixture of individuals from many ethnic groups and racial backgrounds. Microbial diversity inside an individual gets impacted by such racial diversity and social interaction. The majority of probiotic advantages are either anecdotal or have scant scientific support. The novel and potential preventative and therapeutic agents for developing customized medicine may be the health‐promoting commensals. These commensals are known as next‐generation probiotics (NGPs), and because of their peculiar traits, uncertain identities, and unique growth needs, study has been challenging. Several in vivo and clinical studies have established the therapeutic value of probiotics in the treatment of lifestyle diseases, though it is confirmed that strains and species of probiotics have a significant impact on these beneficial effects on health, and their efficacy is altered by various factors like their origin (human or non‐human), race and ethnicity, geographical location, etc. Hence, the goal of the current study was to come up with indigenous LAB strains having the potential to be used as probiotics having higher antioxidant properties. Isolation was performed for 120 different locally obtained dairy products. A total of 224 colonies were screened for primary and secondary probiotic attributes. Only 6 potential probiotic strains were found to have all the probiotic attributes.

All these isolates showed growth at acidic pH from 1.5 to 2 for at least two hours, survived 2% bile salt, and demonstrated > 75% cell autoaggregation properties. In addition, their antioxidant potential was determined by three different methods, namely ABTS, DPPH, and FRAP methods. For all three methods, *L. rhamnosus* was taken as a positive control that showed 85.61%, 39.56%, and 78.18% reduction of free radicals as determined by the ABTS, DPPH, and FRAP methods, respectively. Compared to this, *Limosilactobacillus fermentum* BAB 7912 demonstrated the highest reduction of ABTS radicals (83.45%), while *Bacillus subtilis* BAB 7918 reduced 29.95% DPPH free radicals and *Bacillus spizizenii* BAB 7915 reduced 80.93% ferric ions as determined by the FRAP method.

Our research outcome has added six new probiotic strains that belong to Bacillus, Lactobacillus, and Enterococcus genus. Compared to previously reported isolates as an outcome of various researches from Gujarat and India, our isolates have shown higher CSH and cell autoaggregation properties, which directly influence the culture's abilities to strongly adhere to gut linings. This will lead to reduction in the pathogenic population and add to healthier gut flora. The isolates reported in the present study also have higher antioxidant potential, thus indicating their ability to render health benefits against lifestyle diseases. The safety assessment of these isolates assures that all six species were safe for consumption by humans. These all results strongly support the potential technological utility of our strains for further research. On the one hand, our research group is working on consortia formulation and its applicability for ameliorating obesity and malnutrition, while the other two researches are parallelly ongoing on nanobiotics and the foodomics approach, wherein nanoparticles have been synthesized using these isolates that are under evaluation for food shelf life extension.

## Author Contributions

**Ishita Modasiya:** formal analysis (equal), investigation (equal), methodology (equal), validation (lead), visualization (lead), writing – original draft (equal). **Vijay Kumar:** conceptualization (lead), data curation (lead), formal analysis (equal), project administration (lead), resources (supporting), supervision (lead), validation (supporting), writing – original draft (equal), writing – review and editing (equal). **Priya Mori:** methodology (supporting), validation (supporting), writing – original draft (supporting). **Hina Maniya:** methodology (equal), validation (equal). **Mehul Chauhan:** methodology (equal), writing – original draft (supporting). **Chand Ram Grover:** formal analysis (supporting), resources (supporting), writing – review and editing (supporting). **Apurba Kumar Sarkar:** resources (equal), writing – review and editing (equal).

## Ethics Statement

This study does not involve any human or animal trials.

## Consent

All listed authors have read the final manuscript and provided their consent for publication in the present form.

## Conflicts of Interest

The authors declare no conflicts of interest.

## Supporting information


Data S1.

## Data Availability

The data that support the findings of this study are available on request from the corresponding author. The data are not publicly available due to privacy or ethical restrictions.
