# Supplementary material for: In Vitro Screening of Bacterial Isolates From Dairy Products for Probiotic Properties and Other Health‐Promoting Attributes
Source: Food Sci Nutr. 2024 Nov 25;12(12):10756–69. doi: 10.1002/fsn3.4537 (PMC11666839; doi:10.1002/fsn3.4537)
Supplement: Supplementary file 1 — Data S1. [file FSN3-12-10756-s001.docx]

**Supplementary data sheet**

| **Table no** | **Content** |
| --- | --- |
| Table 1 | Results of Microscopic Observation, Micrometry, catalase and oxidase test of selected isolates with standard strains *Lactiplantibacillus plantarum* NCDC 347, *Lacticaseibacillus rhamnosus* NDRI 184 |
| Table 2 | Results of Biochemical characterization of selected isolates with standard strains *Lactiplantibacillus plantarum* NCDC 347, *Lacticaseibacillus rhamnosus* NDRI 184 |
| Table 3 | Cell autoaggregation and cell surface hydrophobicity of isolates with standard strains *Lactiplantibacillus plantarum* NCDC 347, *Lacticaseibacillus rhamnosus* NDRI 184 |
| Table 4 | Antioxidant potential of isolates (FRAP, ABTS, DPPH) with standard strains *Lactiplantibacillus plantarum* NCDC 347, *Lacticaseibacillus rhamnosus* NDRI 184 |
| Table 5 | Selected lactic cultures list on the basis of secondary screening |

Table 1: Results of Microscopic Observation, Micrometry, catalase and oxidase test of selected isolates with standard strains *Lactiplantibacillus plantarum* NCDC 347, *Lacticaseibacillus rhamnosus* NDRI 184

| Isolates | Gram Staining | Negative Staining | Oxidase test | Catalase test |
| --- | --- | --- | --- | --- |
| *Lactiplantibacillus plantarum* NCDC 347 | + | Rod | - | - |
| *Lacticaseibacillus rhamnosus* NDRI 184 | + | Rod | - | - |
| *Bacillus spizizenii* BAB 7915 | + | Rod | - | - |
| *Bacillus subtilis* BAB 7911 | + | Rod | - | - |
| *Bacillus subtilis* BAB 7918 | + | Rod | - | - |
| *Limosilactobacillus fermentum* BAB 7912 | + | Rod | - | - |
| *Enterococcus faecalis* BAB 7913 | + | Cocci | - | - |
| *Enterococcus faecium* BAB 7914 | + | Cocci | - | - |

(+) - Positive, (-) – Negative

Table 2: Results of Biochemical characterization of selected isolates with standard strains *Lactiplantibacillus plantarum* NCDC 347, *Lacticaseibacillus rhamnosus* NDRI 184

| Isolates | Indole | Methyl-Red | Voges-Proskauer | Citrate Utilization | Urease | TSI | Haemolytic |
| --- | --- | --- | --- | --- | --- | --- | --- |
| *Lactiplantibacillus plantarum* NCDC 347 | - | + | + | - | - | + | - |
| *Lacticaseibacillus rhamnosus* NDRI 184 | - | + | + | - | - | + | - |
| *Bacillus spizizenii* BAB 7915 | - | - | + | + | - | + | - |
| *Bacillus subtilis* BAB 7911 | - | - | - | + | - | + | - |
| *Bacillus subtilis* BAB 7918 | - | - | - | + | - | + | - |
| *Limosilactobacillus fermentum* BAB 7912 | - | + | + | - | - | + | - |
| *Enterococcus faecalis* BAB 7913 | - | + | + | - | - | + | - |
| *Enterococcus faecium* BAB 7914 | - | - | - | + | - | + | - |

TSI- Triple sugar ion, (+) - Positive, (-) - Negative

Table 3: Cell autoaggregation and cell surface hydrophobicity of isolates with standard strains *Lactiplantibacillus plantarum* NCDC 347, *Lacticaseibacillus rhamnosus* NDRI 184

| Isolates | Auto autoaggregation (%) (5h) | Hydrophobicity (%) | |
| --- | --- | --- | --- |
|  |  | Xylene | Toluene |
| *Lactiplantibacillus plantarum* NCDC 347 | 98.7±1.0 | 64.1±2.4 | 42.9±2.2 |
| *Lacticaseibacillus rhamnosus* NDRI 184 | 98.9±0.2 | 70.1±5.1 | 46.5±4.0 |
| *Bacillus spizizenii* BAB 7915 | 86.1±0.2 | 62.1±1.9 | 42.3±8.5 |
| *Bacillus subtilis* BAB 7911 | 81.0±2.8 | 88.8±0.6 | 75.3±3.4 |
| *Bacillus subtilis* BAB 7918 | 87.5±0.5 | 79.2±3.5 | 66.5±8.6 |
| *Limosilactobacillus fermentum* BAB 7912 | 89.7±0.5 | 61.2±2.6 | 58.0±2.0 |
| *Enterococcus faecalis* BAB 7913 | 88.7±0.9 | 70.9±1.4 | 67.9±1.6 |
| *Enterococcus faecium* BAB 7914 | 98.3±0.0 | 90.7±1.6 | 96.4±0.1 |

Results are presented as means ± SD from three independent experiments.

Table 4: Antioxidant potential of isolates (FRAP, ABTS, DPPH) with standard strains *Lactiplantibacillus plantarum* NCDC 347, *Lacticaseibacillus rhamnosus* NDRI 184

| Isolates | Reducing power (%) of FRAP | % reduction of ABTS | % reduction of DPPH |
| --- | --- | --- | --- |
| *Lactiplantibacillus plantarum* NCDC 347 | 65.40±0.41 | 85.16±0.92 | 39.69±4.63 |
| *Lacticaseibacillus rhamnosus* NDRI 184 | 78.18±0.08 | 85.61±0.45 | 39.56±0.23 |
| *Bacillus spizizenii* BAB 7915 | 80.93±0.12 | 78.33±0.52 | 16.40±1.34 |
| *Bacillus subtilis* BAB 7911 | 79.67±0.07 | 78.02±0.92 | 7.75±0.70 |
| *Bacillus subtilis* BAB 7918 | 75.96±0.18 | 55.72±0.92 | 29.95±0.40 |
| *Limosilactobacillus fermentum* BAB 7912 | 80.26±0.05 | 83.45±0.39 | 26.46±1.75 |
| *Enterococcus faecalis* BAB 7913 | 75.78±0.05 | 42.09±0.94 | 20.66±0.73 |
| *Enterococcus faecium* BAB 7914 | 78.53±0.05 | 60.02±0.35 | 21.61±0.95 |

Results are presented as means ± SD from three independent experiments.

Table 5: Selected lactic cultures list on the basis of secondary screening

| Lactic isolates | Acid tolerance (pH) | Bile tolerance (%) | Cell autoaggregation (%) | Cell surface hydrophobicity (Xylene) (%) | Cell surface hydrophobicity (Toluene) (%) |
| --- | --- | --- | --- | --- | --- |
| *Lactobacillus plantarum* | 1.5 | 2 | 98.7±1.0 | 64.1±2.4 | 42.9±2.2 |
| *Lactobacillus rhamnosus* | 1.5 | 2 | 98.9±0.2 | 70.1±5.1 | 46.5±4.0 |
| PIG5CI | 2 | 2 | 86.1±0.2 | 62.1±1.9 | 42.3±8.5 |
| PIG3IR | 1.5 | 2 | 86.9±0.8 | 64.2±1.6 | 73.6±4.9 |
| PIG6IR | 2 | 2 | 87.5±0.5 | 79.2±3.5 | 66.5±8.6 |
| PIB9SR | 2 | 2 | 86.44±0.3 | 78.36±2.5 | 90.26±0.3 |
| PIB9M | 2 | 2 | 81.1±2.9 | 88.8±0.7 | 75.3±3.4 |
| PIB9CR | 2 | 2 | 81.3±1.4 | 91.1±0.8 | 88.0±4.1 |
| PIB10CR | 1.5 | 2 | 84.7±2.8 | 84.9±2.0 | 76.4±4.3 |
| PIB10MR | 2 | 2 | 88.0±1.3 | 88.6±3.8 | 88.2±2.1 |
| PIB13MR | 1.5 | 2 | 87.3±0.5 | 71.2±0.4 | 46.5±3.6 |
| PIB14TR | 3 | 2 | 88.7±0.9 | 70.9±1.4 | 67.9±1.6 |
| PIB12FI | 1.5 | 2 | 81.4±1.2 | 51.6±1.4 | 65.9±2.1 |
| PIB12RB | 1.5 | 2 | 89.7±0.5 | 61.2±2.6 | 58.0±2.0 |
| PIM8CR | 1.5 | 2 | 83.6±1.3 | 84.6±1.4 | 78.0±2.0 |
| PIM9CR | 2 | 2 | 88.1±2.1 | 89.2±0.8 | 90.4±0.4 |
| PIM9FI | 1.5 | 2 | 88.7±2.0 | 89.8±1.9 | 89.0±1.7 |
| PIM10FI | 2 | 2 | 87.4±2.0 | 91.6±0.9 | 84.7±0.6 |
| PIC5CR | 1.5 | 2 | 88.0±0.4 | 90.8±0.7 | 88.8±1.4 |
| PIC20SC | 1.5 | 2 | 98.6±0.1 | 80.9±5.7 | 95.1±0.1 |
| PIC20SY | 2 | 2 | 86.6±0.3 | 77.4±0.6 | 93.7±0.6 |
| PIC22IF | 2 | 2 | 98.9±0.1 | 84.9±0.2 | 90.0±1.2 |
| PIC22RI | 1.5 | 2 | 98.3±0.0 | 90.7±1.6 | 96.4±0.1 |
| PIC23R | 1.5 | 2 | 99.4±0.0 | 95.8±0.1 | 95.2±0.4 |
| PIY1RC | 1.5 | 2 | 86.2±0.9 | 76.3±2.8 | 92.9±0.3 |
